# Electrohydraulic lithotripsy using double-balloon enteroscopy for removal of true enterolith in a patient with unknown ileal stenosis

**DOI:** 10.1055/a-2408-9958

**Published:** 2024-09-19

**Authors:** Ryo Jimbo, Haruki Saito, Yuki Satake, Manabu Takeuchi, Shuji Terai

**Affiliations:** 1 38384 Gastroenterology, Nagaoka Red Cross Hospital, Nagaoka, Japan; 2 Gastroenterology and Hepatology, Niigata University Graduate School of Medical and Dental Sciences, Niigata, Japan

A 71-year-old woman with no prior gastrointestinal diseases presented with lower abdominal pain and vomiting. Abdominal X-ray and computed tomography revealed a 2.6-cm enterolith in the ileum, causing intestinal obstruction ( Fig. 1 , Fig. 2 ). Conservative treatment relieved the obstruction; however, the enterolith persisted. The patient opted for endoscopic treatment over surgery alone. Transanal double-balloon enteroscopy (DBE) using an EI-580BT endoscope (Fujifilm, Tokyo, Japan) revealed stenosis in the distal ileum ( Fig. 3 ) and an enterolith located on the proximal side of the stenosis. The enterolith was excessively hard and could not be crushed using a crushing catheter. Therefore, it was fragmented using electrohydraulic lithotripsy (EHL) and removed using a loop net ( Fig. 4 , Video 1 ). The enterolith was composed of calcium oxalate, indicating that it was a true enterolith ( Fig. 5 ).

**Fig. 1:**
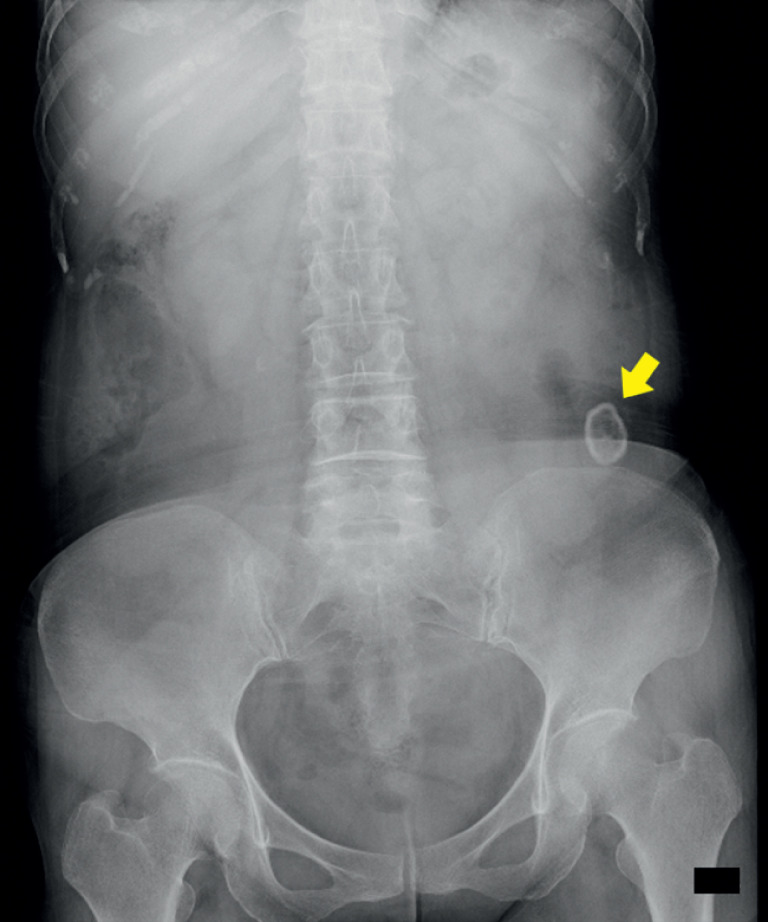
Abdominal X-ray showing a 2.6-cm stone located in the left lower part of the abdomen (yellow arrow).

**Fig. 2:**
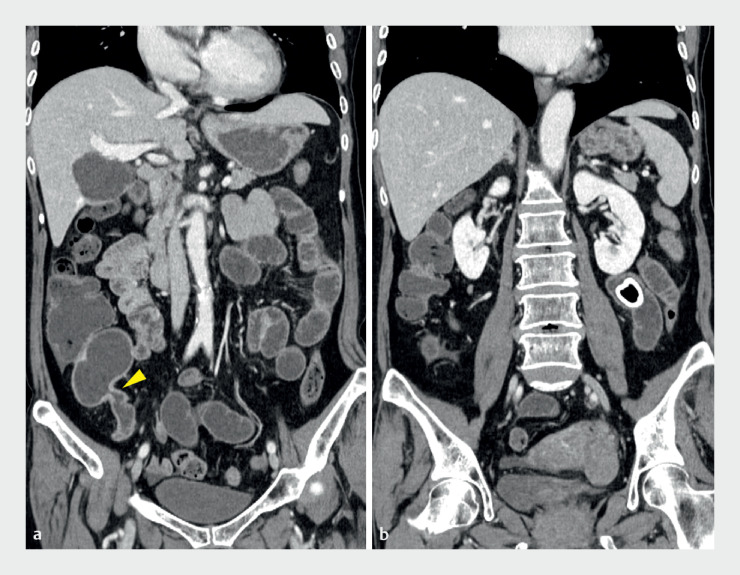
**a, b** Coronal computed tomography revealing stenosis (arrowhead) of the distal ileum ( **a** ) and a 2.6-cm, round hyperdense stone in the ileum ( **b** ).

**Fig. 3:**
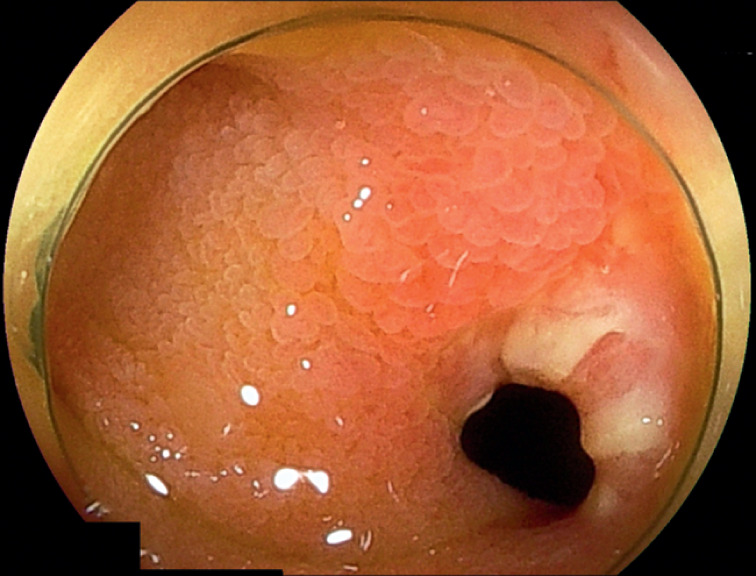
Enteroscopic image showing stenosis of the distal ileum, without findings suggestive of other diseases such as Crohn’s disease or intestinal tuberculosis.

**Fig. 4:**
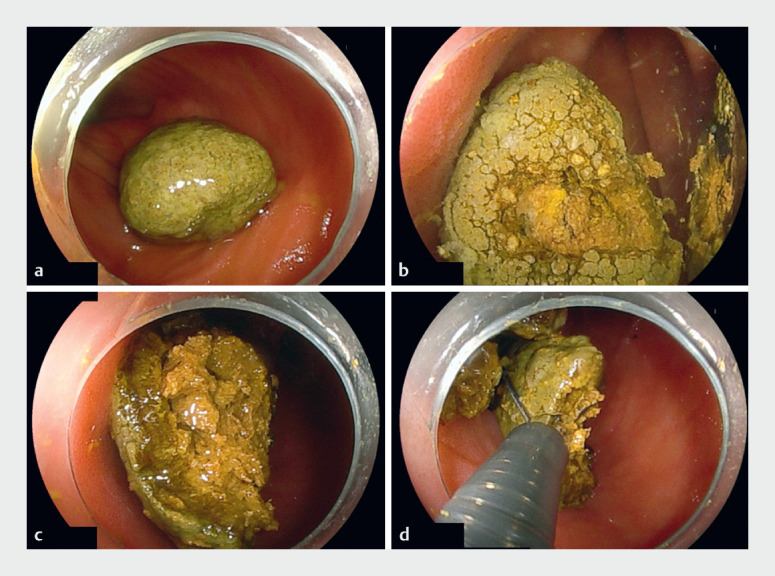
**a** The enterolith was observed on the proximal side of the ileal stenosis. **b** Appearance of the enterolith during electrohydraulic lithotripsy (EHL). **c** The enterolith was successfully crushed into small fragments by EHL. **d** The enterolith fragments were crushed into even smaller fragments using a crushing catheter.

**Fig. 5:**
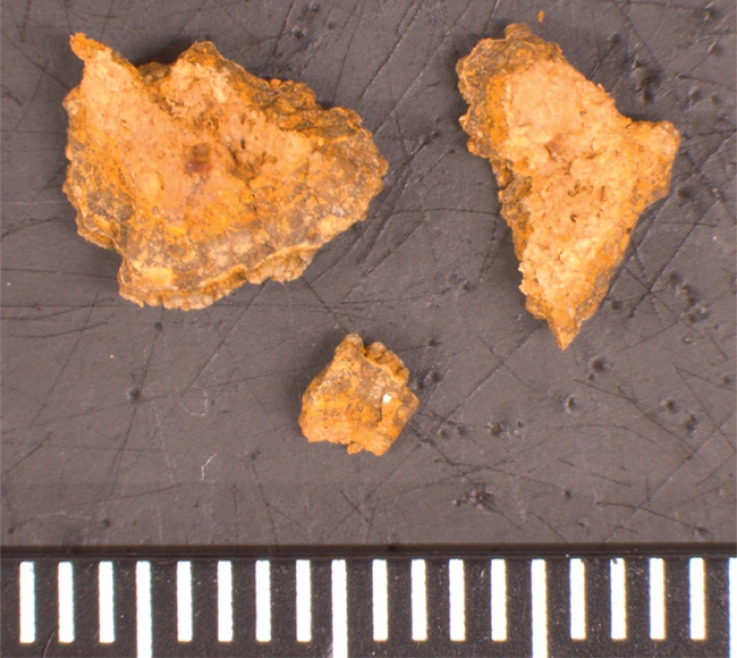
Appearance of the crushed enterolith.

Treatment of an ileal enterolith using electrohydraulic lithotripsy (EHL) with transanal double-balloon enteroscopy (DBE).Video 1

True enteroliths are formed in the small intestine due to intestinal fluid stasis. However, the present case was rare as the patient had ileal stenosis and developed an enterolith despite the absence of underlying diseases, such as Crohn’s disease and intestinal tuberculosis 1 . Endoscopic treatment of enteroliths includes snare lithotripsy 2 , laser lithotripsy 3 , and EHL; however, surgery is the most commonly performed treatment. A previous study on the removal of true enteroliths using DBE demonstrated that EHL is effective 4 , indicating that true enteroliths are often excessively hard and cannot be crushed using other devices because of their predominant calcium composition 5 . EHL is a minimally invasive alternative to surgery for true enteroliths, particularly hard enteroliths.

In summary, we report the treatment of a true enterolith using EHL with transanal DBE in a patient with previously unknown ileal stenosis, avoiding the need for surgery.

Endoscopy_UCTN_Code_TTT_1AP_2AD

## Notes

Endoscopy E-Videos https://eref.thieme.de/e-videos
*E-Videos* is an open access online section of the journal *Endoscopy* , reporting on interesting cases and new techniques in gastroenterological endoscopy. All papers include a high-quality video and are published with a Creative Commons CC-BY license. Endoscopy E-Videos qualify for HINARI discounts and waivers and eligibility is automatically checked during the submission process. We grant 100% waivers to articles whose corresponding authors are based in Group A countries and 50% waivers to those who are based in Group B countries as classified by Research4Life (see: https://www.research4life.org/access/eligibility/ ). This section has its own submission website at https://mc.manuscriptcentral.com/e-videos .
